# Psychosocial stratification of antenatal indicators to guide population-based programs in perinatal depression

**DOI:** 10.1186/s12884-021-03722-8

**Published:** 2021-04-06

**Authors:** E. D. John Eastwood, Andy Wang, Sarah Khanlari, Alicia Montgomery, Jean Yee Hwa Yang

**Affiliations:** 1 Child & Family Clinical Services, Community Health, Sydney Local Health District, 24 Liverpool Road, Croydon, NSW 2132 Australia; 2 Clinical Services Integration and Population Health, Sydney Local Health District, Camperdown, Australia; 3 Sydney Institute for Women, Children and their Families, Sydney Local Health District, Camperdown, Australia; 4 Faculty of Medicine and Health, University of Sydney, Sydney, Australia; 5 Department of Anaesthesia, Royal Prince Alfred Hospital, Sydney Local Health District, Camperdown, Australia; 6 School of Mathematics and Statistics, The University of Sydney, Sydney, Australia; 7 Charles Perkins Centre, The University of Sydney, Sydney, Australia

**Keywords:** Perinatal, Depression, Stratification, Integrated care, Latent class analysis

## Abstract

**Background:**

There is increasing awareness that perinatal psychosocial adversity experienced by mothers, children, and their families, may influence health and well-being across the life course. To maximise the impact of population-based interventions for optimising perinatal wellbeing, health services can utilise empirical methods to identify subgroups at highest risk of poor outcomes relative to the overall population.

**Methods:**

This study sought to identify sub-groups using latent class analysis within a population of mothers in Sydney, Australia, based on their differing experience of self-reported indicators of psychosocial adversity. This study sought to identify sub-groups using latent class analysis within a population of mothers in Sydney, Australia, based on their differing experience of self-reported indicators of psychosocial adversity. Subgroup differences in antenatal and postnatal depressive symptoms were assessed using the Edinburgh Postnatal Depression Scale.

**Results:**

Latent class analysis identified four distinct subgroups within the cohort, who were distinguished empirically on the basis of their native language, current smoking status, previous involvement with Family-and-Community Services (FaCS), history of child abuse, presence of a supportive partner, and a history of intimate partner psychological violence. One group consisted of socially supported ‘local’ women who speak English as their primary language (Group L), another of socially supported ‘migrant’ women who speak a language other than English as their primary language (Group M), another of socially stressed ‘local’ women who speak English as their primary language (Group Ls), and socially stressed ‘migrant’ women who speak a language other than English as their primary language (Group Ms.). Compared to local and not socially stressed residents (L group), the odds of antenatal depression were nearly three times higher for the socially stressed groups (Ls OR: 2.87 95%CI 2.10–3.94) and nearly nine times more in the Ms. group (Ms OR: 8.78, 95%CI 5.13–15.03). Antenatal symptoms of depression were also higher in the not socially stressed migrant group (M OR: 1.70 95%CI 1.47–1.97) compared to non-migrants. In the postnatal period, Group M was 1.5 times more likely, while the Ms. group was over five times more likely to experience suboptimal mental health compared to Group L (OR 1.50, 95%CI 1.22–1.84; and OR 5.28, 95%CI 2.63–10.63, for M and Ms. respectively).

**Conclusions:**

The application of empirical subgrouping analysis permits an informed approach to targeted interventions and resource allocation for optimising perinatal maternal wellbeing.

## Background

The association of adversity during pregnancy with poor pregnancy and childbirth outcomes is well established [1–3]. The antenatal stressors are broadly related to the intrauterine environment (e.g. nutrition; maternal stress; exposure to smoking, drugs and alcohol), psychosocial experience (e.g. interpersonal violence; loneliness; anxiety and depression), and socioeconomic context (e.g. low income; class; migrant; unemployment; education; housing) a mother has experienced and is currently experiencing [4]. The public health importance of this perinatal adversity is related to its demonstrated impact on multiple domains of childhood and adult outcomes across the life course [5, 6]. There is an increasing understanding of the impact of exposure to adverse childhood experiences (such as child maltreatment and exposure to domestic violence), on health and well-being outcomes across the life course. There is an intergenerational impact and a strong dose-response relationship between exposure to adversity and poor health outcomes, including depression, anxiety, substance use, sexually transmitted diseases, suicide attempts, and a range of chronic diseases [7]. Furthermore, there is an association between adverse childhood experiences and increased risk of parental mental illness and substance abuse in pregnancy [8], and there is increasing interest in the role that maternal mental health plays in the intergenerational transmission of experienced adversity [9, 10].

Significant relationships have been demonstrated between maternal depressive symptoms, their family and social circumstances, factors relating to community integration and ethnicity, and history of professional psychosocial support received [11, 12]. In the antenatal and postpartum periods, increased levels of social support provision have a positive effect on decreasing depression risk [12, 13]. Antenatal events and social circumstances, such as disease during pregnancy, family dissatisfaction, or social isolation, have also been identified as risk factors for postnatal depression [14, 15]. Maternal antenatal and postnatal depressive symptoms are strongly associated with numerous adverse perinatal outcomes including preterm delivery and low birth weight [13, 16–20].

Psychosocial assessment during pregnancy can identify both risk and protective factors for the development of perinatal mood disorders. The New South Wales (NSW) Safe Start Policy [21] is a universally delivered programme for publicly booked pregnant women in the state of NSW, Australia. The programme incorporates antenatal and postnatal psychosocial assessment and the risk factors identified are used to organise further assessment and intervention. The Safe Start risk stratification framework was developed following a rigorous analysis of literature and expert policy advice. We are not aware of previous empirical studies that have sought to utilise psychosocial information obtained via Safe Start screening to quantify the sub-populations at risk using latent class analysis or other cluster analysis approaches. The study reported here is part of a translational psychosocial epidemiology study of perinatal adversity in the Sydney Local Health District (SLHD) and South Western Sydney Local Health District (SWSLHD) in Australia. In Sydney, these two districts cover 52% of the metropolitan area, with an estimated population of 1.6 million people of different cultural backgrounds [22, 23]. A number of maternal and child health services are provided to all communities across both districts, including those with socioeconomically disadvantaged populations [22, 23]. In this study, we examined whether maternal sub-groups can be identified on the basis of their varying experiences of adversity, and whether the risk of antenatal and postnatal depressive symptoms differs between sub-groups, to inform maternal and child health service system redesign in Sydney.

## Methods

**Fig. 1:**
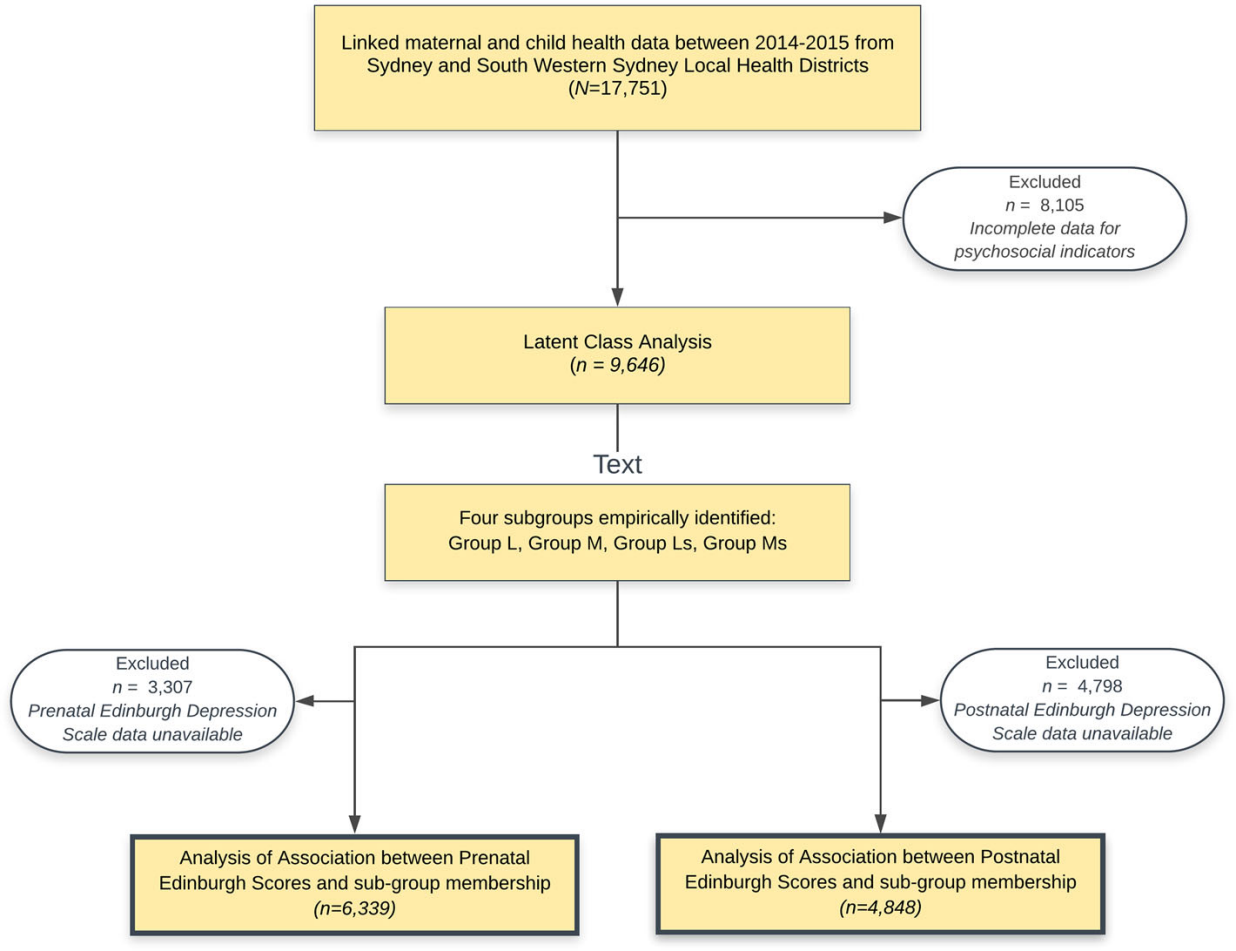
Study flow chart

This study utilised antenatal and postnatal data extracted from maternal and child health electronic medical records in the Sydney Local Health District (SLHD) and South Western Sydney Local Health District (SWSLHD), with ethical approval obtained from both health districts. The overall data sources used in this study have been described elsewhere [17, 24]. For this study, linked retrospective maternal and child health data of all live births in public health facilities in the Sydney Local Health District (SLHD) and South Western Sydney Local Health District (SWSLHD) between 2014 and 2015 (*N* = 17,751) were available. These data were routinely collected by qualified midwives as part of standard care provided to women during pregnancy and the postnatal period (within 6 weeks of birth). Non-English speaking pregnant women were provided with translated versions of the EPDS where available, produced by the New South Wales Multicultural Health Communication Service [25]. Alternatively, women completed the English version of the EPDS through accredited interpreters. Of the 17,751 medical records available, a total of 8105 participants were excluded due to incomplete information on psychosocial indicators that were mandatory to determine stratification class membership, with *n* = 9646 mothers included in subsequent analysis. These women had complete psychosocial data collected during their first antenatal encounter to enable assignment of group membership according to psychosocial stratification using latent class analysis, in addition to an EPDS score from an antenatal booking visit (*n* = 6339), and/or a postnatal EPDS score (*n* = 4848), as shown in Fig. 1.

Demographic variables utilised in this study included maternal age, gestational age at the first visit, pre-pregnancy BMI, smoking status, whether English was the first language or spoken at home, country of birth, and Indigenous status. Psychosocial indicators were extracted from the electronic medical record to reflect the four critical antenatal psychosocial health assessment domains described in the ALPHA model [26], which include family factors, maternal factors, substance use, and family violence. Available social indicators pertaining to family factors included presence or absence of a partner, report of having a supportive partner, previous involvement of statutory child protection agencies - Family-and-Community-Services (FaCS) or Out-of-Home-Care services (OoHC), and socioeconomic status. Socioeconomic status was calculated in accordance with the Australian Bureau of Statistics Socio-Economic Index for Areas [27], based on the mother’s current residential address. Decile of socioeconomic status was categorised into High, Middle and Low groups (top 10%, middle percent and bottom 10% of the population respectively). Available social indicators pertaining to maternal factors included thoughts on history of self-harm, history of child abuse and history of physical or psychological intimate partner violence. Individuals in this study were stratified into mutually exclusive subpopulations using latent class modelling on the basis of the above psychosocial indicator variables. Then, in a two-step approach, covariate analyses examined for associations between subgroup membership and EPDS scores obtained in the prenatal and postnatal periods separately. The EPDS is a widely-used, multi-dimensional measures of maternal symptoms of anxiety [28, 29] and depression [30], with a total possible score of 30. In the present study, EPDS scores used to indicate suboptimal maternal mental wellbeing were selected based on previously published studies [31, 32] and the current Australian endorsed guidelines on improving mental health outcomes for parents and infants [33]. For the purpose of this study, an EPDS of ≥9 was used as a binary variable to indicate suboptimal maternal mental health, in keeping with previous research indicating that an EPDS score of 9–12 is indicative of clinically relevant maternal dysphoria [34], whilst an EPDS of ≥13 is predictive of probable major depression [35, 36]. The EPDS has been validated across a range of cultures [37] and is superior to unstructured routine assessment in identifying indicators of suboptimal maternal mental health both internationally and in Australia [38].

### Statistical analyses

Latent class analysis (LCA) is an empirical approach to subgroup identification that classifies individuals into distinct categories based on differing patterns of ‘indicator’ variables, such that individuals within a group are more similar than individuals between groups [39]. In this study, LCA was conducted to identify subgroups within the overall population of women on the basis of varying experiences of adversity. The indicator variables used to identify latent classes in this analysis include English as a second language or born overseas, English spoken at home, current smoking status, family known to Family-and-Community Services (FaCS), smoking status, history of child abuse, having a supportive partner, history of intimate partner psychological violence, maternal age group, BMI, reported alcohol use, late first antenatal visit (defined as greater than 20 weeks’ gestation), SEIFA of residence, having a partner, prior involvement with Out-Of-Home-Care services (OOHC), and known physical domestic violence.

Goodness-of-fit statistics were then used to identify the optimal model and most likely number of classes to describe the underlying ‘class structure’ in this analysis via scree plot (including the log likelihood ratio, with higher values supporting models of better fit, and the Bayesian Information Criterion (BIC) and Akaike Information Criterion (AIC), with smaller values indicating better model fit). As the context of our study involves different social, ethnic and disadvantaged populations, we will henceforth refer to the latent classes as ‘groups’ to avoid unintentional negative connotations associated with the term ‘class.’ After identification of the optimal underlying latent class structure based on all included indicator variables, a swap-stepwise latent class model comparison approach was used to select the most informative variables (indicators) that characterise specific subgroup membership [40]. This is achieved by discarding those that are redundant (correlated), with intermittent swapping of variables in step-wise fashion, and comparing models with and without the assumption of independence between indicators.

Finally, risk of suboptimal perinatal mental health was assessed by odds ratio within each stratified subpopulation group, at both the first antenatal visit and the postnatal visit (within 6 weeks postpartum). Multivariate logistic regression modelling was performed to adjust for potentially confounding variables (represented by those that were not used as indicators in the latent class analysis as described above). All statistical analyses were performed using R.

## Results

The cohort’s demographic and psychosocial characteristics are displayed in Table 1.

**Table 1:** Mothers in the Sydney and South West Sydney Local Health Districts (*N* = 9646)

	Group L^a^ n (%)	Group Ls^b^ n (%)	Group M^c^ n (%)	Group Ms.^d^ n (%)
	4573 (47%)	353 (4%)	4622 (48%)	98 (1%)
***Demographic Characteristics***				
Age at 1st antenatal visit, *mean (sd)*	29.93 (5.66)	28.36 (6.16)	31.12 (4.97)	32.43 (5.90)
20–39 years old	4221 (92.3%)	319 (90.37%)	4372 (94.59%)	86 (87.76%)
≥ 40 years old	225 (4.92%)	14 (3.97%)	230 (4.98%)	9 (9.18%)
< 20 years old	127 (2.78%)	20 (5.67%)	20 (0.43%)	3 (3.06%)
Pre-pregnancy BMI in *kg*/*m*^2^, *mean (sd)*	25.75 (6.13)	25.61 (6.40)	23.71 (4.83)	24.80 (5.37)
Underweight	186 (4.19%)	37 (10.88%)	350 (7/76%)	7 (7.29%)
Normal weight ()	2260 (50.86%)	145 (42.65%)	2723 (60.36%)	52 (54.17%)
Overweight	1083 (24.37%)	80 (23.53%)	969 (21.48%)	23 (23.96%)
Obese	915 (20.59%)	78 (22.94%)	469 (10.40%)	14 (14.58%)
Born overseas or ESL				
No	4407 (96.37%)	350 (99.15%)	0 (0.00%)	5 (5.10%)
Yes	166 (3.63%)	3 (0.85%)	4622 (100.00%)	93 (94.90%)
Speaking English at home				
Yes	4486 (98.10%)	353 (100%)	2357 (51.00%)	60 (61.22%)
No	87 (1.90%)	0 (0%)	2265 (49.00%)	38 (38/78%)
Indigenous Status				
No	4426 (96.85%)	286 (81.48%)	4615 (99.94%)	97 (98.98%)
Yes	144 (3.15%)	65 (18.52%)	3 (0.06%)	1 (2.21%)
Socio-Economic Index for Areas				
Low	830 (18.25%)	114 (32.48%)	1765(40.33%)	55 (56.70%)
Medium	3420 (75.21%)	230 (65.53%)	2469 (56.42%)	37 (38.14%)
High	297 (6.53%)	7 (1.99%)	142 (3.24%)	5 (5.15%)
***Family Factors***				
Has a partner				
No	191 (4.21%)	106 (30.37%)	111 (2.43%)	26 (26.80%)
Yes	4345 (95.79%)	243 (69.63%)	4459 (97.57%)	71 (97.57%)
Supportive Partner				
No	65 (1.42%)	98 (27.76%)	110 (2.38%)	42 (42.86%)
Yes	4508 (98.58%)	255 (72.24%)	4512 (97.62%)	56 (57.14%)
Known to Family-and-Community Services				
No	4573 (100.00%)	164 (46.46%)	4622 (100.00%)	62 (63.27%)
Yes	0 (00.00%)	189 (53.54%)	0 (0.00%)	36 (36.73%)
Known to Out-of-Home-Care Services				
No	3285 (97.42%)	196 (67.12%)	3465 (97.58%)	64 (84.21%)
Yes	87 (2.58%)	96 (32.88%)	86 (2.42%)	12 (15.79%)
***Maternal Factors***				
Thoughts of self-harm				
No	4168 (98.96%)	290 (92.36%)	4192 (98.94%)	73 (85.88%)
Yes	44 (1.04%)	24 (7.64%)	45 (1.06%)	12 (14.12%)
Gestational age at 1st antenatal visit, *mean (sd)*	12.57 (7.37)	14.46 (8.36)	12.49 (7.54)	15.55 (8.70)
<20 weeks	3749 (83.20%)	255 (74.13%)	3760 (82.47%)	62 (63.27%)
≥20 weeks	757 (16.80%)	89 (25.87%)	799 (17.53%)	36 (36.73%)
***Substance Use***				
Smoking status				
No	4011 (87.71%)	104 (29.46%)	4589 (99.29%)	84 (85.71%)
Yes	562 (12.29%)	249 (70.54%)	33 (0.71%)	14 (14.29%)
Alcohol use				
No	4407 (98.98%)	325 (92.59%)	4533 (99.32%)	93 (98.94%)
Yes	91 (2.02%)	26 (7.41%)	31 (0.68%)	1(1.06%)
***Family Violence***				
History of Child Abuse				
No	4145 (90.64%)	166 (47.03%)	4585 (99.20%)	63 (64.29%)
Yes	428 (9.36%)	187 (52.97%)	37 (0.80%)	35 (35.71%)
Intimate Partner Physical Violence				
No	4547 (99.56%)	293 (83.00%)	4567 (99.22%)	74 (75.51%)
Yes	20 (0.44%)	60 (17.00%)	36 (0.78%)	24 (24.49%)
Intimate Partner Psychological Violence				
No	4573 (100.00%)	297 (84.14%)	4608 (99.70%)	52 (53.06%)
Yes	0 (0.00%)	56 (15.86%)	14 (0.30%)	46 (46.94%)
***Edinburgh Postnatal Depression Scales (EPDS)***				
Antenatal EPDS, *median (IQR)*	3 (5)	7 (7)	5 (6)	11 (10.5)
EPDS < 9	3762 (86.66%)	198 (60.37%)	3378 (78.41%)	33 (37.93%)
EPDS ≥ 9	673 (13.34%)	130 (39.63%)	930 (21.59%)	54 (62.07%)
Postnatal EPDS, *median (IQR)*	3 (4)	3 (5)	3 (5)	6 (7)
EPDS < 9	3139 (90.23%)	205 (87.23%)	2880 (86.41%)	44 (47.69%)
EPDS ≥ 9	340 (9.77%)	30 (12.77%)	453 (13.59%)	21 (32.31%)

^a^Socially supported ‘local’ women who speak English as their primary language (Group L)

^b^Socially supported ‘migrant’ women who speak a language other than English as their primary language (Group M)

^c^Socially stressed ‘local’ women who speak English as their primary language (Group Ls)

^d^Socially stressed ‘migrant’ women who speak a language other than English as their primary language (Group Ms.)

### Latent class analysis

**Fig. 2:**
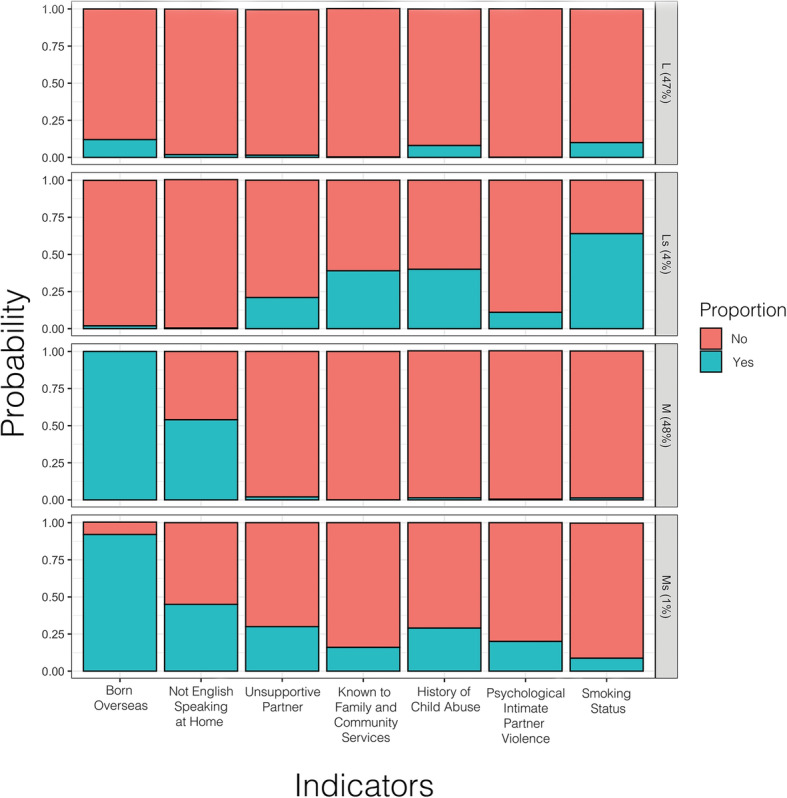
Scree plot for model fits with 1, 2, 3, 4, 5, and 6 latent class models respectively

Latent class analysis of all included indicator variables suggested a three or four class model was most likely, as visualised via scree plot (Fig. 2). After examining the latent class structures for both the three- and four-class models, the four-class latent structure was considered to be the most valid on the basis of community experience. It consists of a subpopulation of women who speak English as their first language (described as ‘local’) (L), and a subpopulation who speak English as a second language (described as ‘migrant’) (M) women, who are either socially supported or socially-stressed (s). Swap-stepwise latent class model comparison then identified the most informative variables (indicators) characterising specific subgroup membership, which include a previous history of involvement with Family-and-Community Services (FaCS), smoking status, a previous history of child abuse, presence of a supportive partner, and history of intimate partner psychological violence. Other indicators that were not found to significantly influence the latent class structure or were redundant (highly correlated with the included indicator variables above), include maternal age group, body mass index, reported alcohol use, late first antenatal visit (defined as greater than 20 weeks’ gestation), SEIFA of residence, having a partner, prior involvement with Out-Of-Home-Care services (OOHC), and known physical domestic violence.

**Fig. 3:**
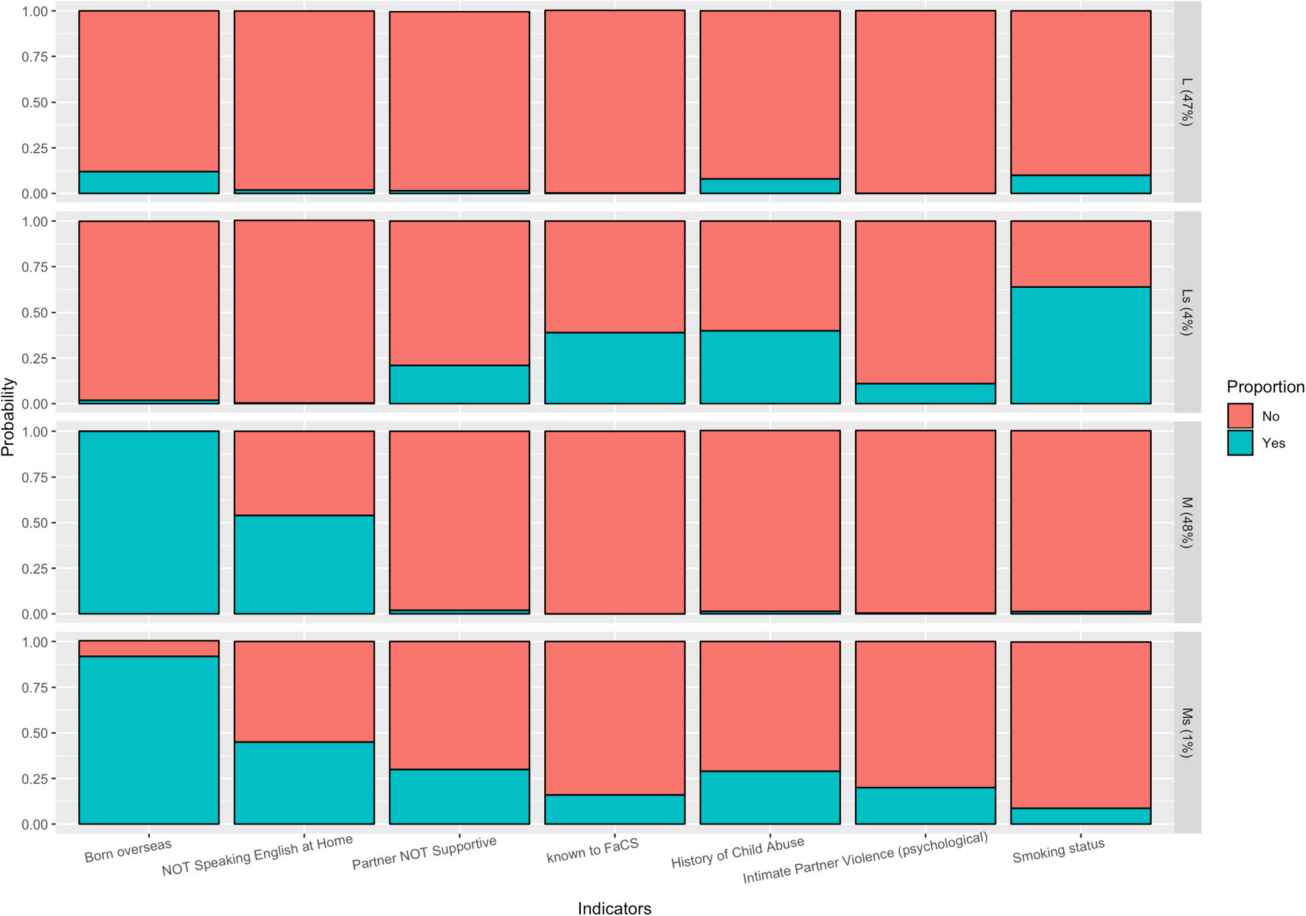
Subpopulation stratification into four groups – Local (L), Stressed Local (Ls), Migrant (M), and Stressed Migrant (Ms.)

The four subpopulation groups identified were: a) a majority group of local Australian residents with minimal social stressors (Group L, 47%, *n* = 4573); b) a small group of socially stressed local residents (Group Ls, 4%, *n* = 353); c) a majority group of migrants with minimal social stressors (Group M, 48%, *n* = 4622); and d) a small socially stressed migrant group (Group Ms., 1%, *n* = 98) (Fig. 3).

### Maternal antenatal depression scores by subgroup

The mean (SD) age of the study participants at the first antenatal visit was 30.5 (SD 5.4) years old. The median EPDS score at the first antenatal visit was 4 (IQR = 6). 14% were considered to exhibit depressive symptoms with a score > 9. There was no EPDS score recorded for 582 women. The prevalence of depression in this cohort was 19.5% overall, and was 13.34%, 39.63%, 21.59% and 62.07% in Groups L, Ls, M and Ms. respectively.

After adjustment, compared to local and not socially stressed residents (L group), the odds of depression were much higher for the socially stressed groups (Ls OR: 2.87 95%CI 2.10–3.94; Ms. OR: 8.78, 95%CI 5.13–15.03), being nearly three times more in the Ls group and nearly nine times more in the Ms. group. Antenatal depressive symptoms were also higher in the not socially stressed migrant group (Ms. OR: 1.70 95%CI 1.47–1.97) compared to non-migrants.

### Maternal postnatal depression scores by subgroup

The median EPDS score at the postnatal visit was 3 (IQR = 5), with 9.1% having an EPDS score of more than 9. The prevalence of depressive symptoms indicated by an EPDS ≥9 in this cohort was 11.9% overall, and 9.77%, 12.77%, 13.59% and 32.31% in Groups L, Ls, M and Ms. respectively, notably lower than the respective groups at the first antenatal visit.

After covariate adjustments, Group M was 1.5 times more likely, while Ms. group was more than five times more likely, to experience suboptimal mental health in the postpartum period compared to Group L (OR 1.50, 95%CI 1.22–1.84; and OR 5.28, 95%CI 2.63–10.63, for M and Ms. respectively).

Table 2 lists univariate and multivariate odds ratios between depression and each of the nine variables in the antenatal period, while Table 3 lists those in the post-natal period.

**Table 2:** Antenatal Period – Univariate estimates vs adjusted estimates

		Univariate Estimates			Multivariate Estimates		
*Variable*	*Level*	*OR*	*OR 95%CI*	*P*	*OR*	*OR 95%CI*	*p*
Group	*L (Ref)*						
	*Ls*	4.08	3.10, 5.37	< 0.0001	2.87	2.10, 3.94	< 0.0001
	*M*	1.63	1.42, 1.87	< 0.0001	1.70	1.47, 1.97	< 0.0001
	*Ms.*	11.04	6.58, 18.51	< 0.0001	8.78	5.13, 15.03	< 0.0001
Age	*20–39*						
	*≥40*	1.18	0.91, 1.54	0.21	1.10	0.84, 1.44	0.51
	*<20*	1.38	0.72, 2.62	0.34	1.29	0.64, 2.60	0.48
Body Mass Index	*Normal (Ref)*						
	*Underweight*	1.14	0.86, 1.53	0.36	0.96	0.71, 1.31	0.81
	*Overweight*	1.20	1.03, 1.40	0.02	1.23	1.05, 1.44	0.01
	*Obese*	1.29	1.08, 1.54	0.004	1.40	1.17, 1.68	0.0002
Consumes Alcohol	*No (Ref)*						
	*Yes*	1.88	1.20, 2.92	0.005	1.70	1.06, 2.71	0.03
Late 1st Antenatal Visit	*No (Ref)*						
	*Yes*	1.02	0.86, 1.21	0.81	0.88	0.74, 1.05	0.1
Partner	*Yes (Ref)*						
	*No*	2.04	1.58, 2.64	< 0.0001	1.41	1.06, 1.88	0.02
History of Physical Domestic Violence	*No (Ref)*						
	*Yes*	5.24	3.46, 7.92	< 0.0001	2.43	1.52, 3.86	0.0002
Previous Out of Home Care Involvement	*No (Ref)*						
	*Yes*	2.16	1.62, 2.87	< 0.0001	1.37	0.99, 1.90	0.06
Socio-Economic Indexes for Areas (SEIFA) Group	*Medium (Ref)*						
	*Low*	1.30	1.13, 1.49	< 0.001	1.08	0.94, 1.26	0.28
	*High*	1.13	0.82, 1.54	0.45	1.25	0.91, 1.74	0.17

**Table 3:** Postnatal Period – Univariate estimates vs adjusted estimates

		Univariate Estimates			Multivariate Estimates		
*Variable*	*Level*	*OR*	*OR 95%CI*	*P*	*OR*	*OR 95%CI*	*p*
Group	*L (Ref)*						
	*Ls*	1.33	0.81, 2.19	0.26	1.25	0.73, 2.16	0.42
	*M*	1.47	1.21, 1.79	< 0.0001	1.50	1.22, 1.84	0.0001
	*Ms.*	5.41	2.79, 10.49	< 0.0001	5.28	2.63, 10.63	< 0.0001
Age	*20–39*						
	*≥40*	0.98	0.66, 1.45	0.91	0.89	0.60, 1.34	0.58
	*<20*	0.46	0.11, 1.91	0.28	0.65	0.16, 2.76	0.56
Body Mass Index	*Normal (Ref)*						
	*Underweight*	0.66	0.40, 1.09	0.10	0.64	0.39, 1.06	0.08
	*Overweight*	1.12	0.89, 1.39	0.331	1.15	0.92, 1.44	0.21
	*Obese*	0.95	0.73, 1.24	0.70	1.04	0.79, 1.37	0.77
Consumes Alcohol	*No (Ref)*						
	*Yes*	1.48	0.78, 2.84	0.23	1.59	0.82, 3.07	0.17
Late 1st Antenatal Visit	*No (Ref)*						
	*Yes*	0.81	0.63, 1.06	0.13	0.77	0.59, 1.01	0.06
Partner	*Yes (Ref)*						
	*No*	1.00	0.63, 1.59	0.99	0.99	0.55, 1.49	0.69
History of Physical Domestic Violence	*No (Ref)*						
	*Yes*	1.81	0.94, 3.49	0.08	1.42	0.68, 2.96	0.35
Previous Out of Home Care Involvement	*No (Ref)*						
	*Yes*	1.35	0.85, 2.14	0.20	1.22	0.75, 2.00	0.42
Socio-Economic Indexes for Areas (SEIFA) Group	*Medium (Ref)*						
	*Low*	1.13	0.92, 1.39	0.23	1.00	0.81, 1.24	0.99
	*High*	0.97	0.61, 1.55	0.90	0.98	0.61, 1.57	0.94

## Discussion

The latent class analysis reported here has identified five clinical indicators that are strongly associated with a mother’s membership of a stressed subpopulation, and her probability of having both antenatal and postnatal depressive symptoms. Using routinely available clinical and demographic antenatal data, women presenting to the antenatal care services were stratified into four groups that closely resembled the local community experience. The two demographic indicators that were empirically identified as determinants of subgroup membership in this study were being born overseas and speaking English at home. The clinical indicators of psychosocial stress that were empirically identified to determine subgroup membership were: a) having a supportive partner, b) known to Family-and-Community Services (child protection agency), c) smoking status, d) mothers’ history of child abuse, and e) known intimate partner psychological violence.

Depressive symptoms were higher among the two socially stressed groups compared to the two not stressed groups. Other psychosocial factors that influenced antenatal depression between subgroups were pre-pregnancy BMI categories (i.e. being overweight or obese), alcohol use, presence or absence of a partner, and if there was known history of physical domestic violence. Our findings are consistent with previous studies that social stressors are associated with perinatal depressive symptoms. In particular, our findings confirm that psychosocial adversity is affecting pregnant women’s mental wellbeing prenatally. An opportunity exists to address this by targeting socially stressed women in the Ls and Ms. groups either prenatally, or early during pregnancy.

As previously reported, the prevalence of postnatal depressive symptoms was higher in the migrant groups than in the local resident’s groups. Importantly, mothers who were members of socially stressed migrant groups were more than five times more likely to experience depressive symptoms (OR: 5.28, 95%CI: 2.63–10.63). Previous studies have suggested that social isolation and a lack of social networks are important determinants of perinatal depressive symptoms for migrants. The interplay between migrant women’s social networks, integration and perinatal depression is complex. Migrants who reside in communities with a predominantly different cultural background to their own have been shown to have higher rates of depression [41–43]. This suggests that migrant women who integrate successfully into their local community, either within a community of their own cultural background or successfully integrate with a different cultural community, will assume the same psychosocial risks for perinatal depression as their local counterparts [44].

In addition, our results show that migrant mothers who were not members of the ‘stressed’ group, were also more likely to have depressive symptoms postnatally than non-migrant mothers in the non-socially stressed group. It might be that these women find themselves more socially isolated and emotionally stressed in the community postpartum, and not fully utilise supportive services for early motherhood. The opportunity thus exists to provide community support services targeting migrants in the early period postpartum.

The study reported here has a number of limitations which we have described in previous analyses of this data set [17, 45–48]. These include limitations associated with data that is obtained from routinely recorded information in maternity and child health medical records, which contains some missing data. In previous studies we have accommodated for missing values via imputation, and previously reported studies found no significant differences when sensitivity analysis was undertaken between imputed and the original dataset. As noted earlier, in this study we excluded those records that had incomplete information. Secondly, we acknowledge that unmeasured variables such as social support, childhood adverse experiences and family structure, may be important for risk stratification in our population of interest. Finally, as previously reported, this study was unable to differentiate mothers with pre-existing clinical depression from those with new-onset perinatal depressive symptoms [46].

The public health importance of perinatal adversity to childhood and adult outcomes has been well described and accepted by the scientific community [5, 6]. There is a significant body of empirical research that has demonstrated that interventions to address perinatal adversity are efficacious in study conditions [49, 50], but when those interventions are taken to scale there is often a failure to achieve expected outcomes. This may be because those interventions were not tested on those with the greatest need, or because interventions are not designed to target, reach and benefit those end users who will benefit most. Systemic population-based approaches are required to identify those women who will benefit most from obstetric and psychosocial interventions. Such approaches will benefit from the development of analytical tools that can be used to improve the coordination, quality, dose and reach of clinical and public health interventions.

## Conclusion

The analysis reported here has demonstrated that it was possible to stratify pregnant women into subpopulations using their demographic and psychosocial characteristics. Membership status within distinct subgroups was highly predictive of both antenatal and postnatal depression. These findings will inform targeted interventions and resource allocation to address maternal wellbeing in the antenatal and immediately postnatal periods in Sydney. Importantly, the study has confirmed the importance of supporting migrant women who are experiencing adversity in the perinatal period.

## Data Availability

The data used for the analysis are accessed in accordance with ethical protocols that only allow unit record information to be released to investigators in the ethics committee submission for study approvals. Please send data requests and queries to South Western Sydney Local Health District Ethics committee. Postal Address: Research and Ethics Office Locked Bag 7103 LIVERPOOL BC NSW 1871 Australia Phone: + 61 (02) 8738 8304; Fax: + 61 (02) 8738 8310; email: research.support@sswahs.nsw.gov.au Sydney Local Health District Ethics committee c/− Research Ethics and Governance Office (REGO) Royal Prince Alfred Hospital Missenden Road CAMPERDOWN NSW 2050 Australia Telephone: + 61 (02) 9515 6766; Facsimile: + 61 (02) 95157176.

## Abbreviations

MatAgeGrp: Maternal age groups
BMI: Body mass index
EtOH: Alcohol
Late1stANV: Late first antenatal visit
SEIFA: Social economic indexes for areas
OOHC: Out of home care
DV.phy: Physical domestic violence
EPDS: Edinburgh Postnatal Depression Scale
NSW: New South Wales
SES: Socio-economic status
SLHD: Sydney Local Health District
SWSLHD: South Western Sydney Local Health District
